# CRISPR-Cas9 Editing of Human Histone Deubiquitinase Gene *USP16* in Human Monocytic Leukemia Cell Line THP-1

**DOI:** 10.3389/fcell.2021.679544

**Published:** 2021-05-31

**Authors:** Iveta Gažová, Lucas Lefevre, Stephen J. Bush, Rocio Rojo, David A. Hume, Andreas Lengeling, Kim M. Summers

**Affiliations:** ^1^The Roslin Institute, University of Edinburgh, Easter Bush, United Kingdom; ^2^Mater Research Institute - University of Queensland, Translational Research Institute, Woolloongabba, QLD, Australia

**Keywords:** histone deubiquitinases, epigenetic modifications, THP-1 cell line, genome editing, macrophage, monocyte, *USP16* gene

## Abstract

USP16 is a histone deubiquitinase which facilitates G2/M transition during the cell cycle, regulates DNA damage repair and contributes to inducible gene expression. We mutated the *USP16* gene in a high differentiation clone of the acute monocytic leukemia cell line THP-1 using the CRISPR-Cas9 system and generated four homozygous knockout clones. All were able to proliferate and to differentiate in response to phorbol ester (PMA) treatment. One line was highly proliferative prior to PMA treatment and shut down proliferation upon differentiation, like wild type. Three clones showed sustained expression of the progenitor cell marker *MYB*, indicating that differentiation had not completely blocked proliferation in these clones. Network analysis of transcriptomic differences among wild type, heterozygotes and homozygotes showed clusters of genes that were up- or down-regulated after differentiation in all cell lines. Prior to PMA treatment, the homozygous clones had lower levels than wild type of genes relating to metabolism and mitochondria, including *SRPRB*, encoding an interaction partner of USP16. There was also apparent loss of interferon signaling. In contrast, a number of genes were up-regulated in the homozygous cells compared to wild type at baseline, including other deubiquitinases (*USP12, BAP1*, and *MYSM1*). However, three homozygotes failed to fully induce *USP3* during differentiation. Other network clusters showed effects prior to or after differentiation in the homozygous clones. Thus the removal of USP16 affected the transcriptome of the cells, although all these lines were able to survive, which suggests that the functions attributed to USP16 may be redundant. Our analysis indicates that the leukemic line can adapt to the extreme selection pressure applied by the loss of USP16, and the harsh conditions of the gene editing and selection protocol, through different compensatory pathways. Similar selection pressures occur during the evolution of a cancer *in vivo*, and our results can be seen as a case study in leukemic cell adaptation. USP16 has been considered a target for cancer chemotherapy, but our results suggest that treatment would select for escape mutants that are resistant to USP16 inhibitors.

## Introduction

The development of a tumorigenic lineage from healthy cells is usually associated with a wide range of genetic changes, including point mutations, small and large deletions and insertions (indels) and chromosomal rearrangements (Vogelstein et al., 2013). A high level of genomic instability in cancer cells allows for novel forms of effector molecules to be produced, but also imposes a genetic load of potentially detrimental mutations. Cancer lineages are heterogeneous with many diverse sublineages arising over time (Heppner, 1984; Vogelstein et al., 2013), and these are subjected to intensive selection pressure as the tumor evolves (Fortunato et al., 2017).

One form of modification in cancer lines is alteration in the epigenetic status of the cells. Addition or removal of repressive marks on histone molecules is a major mechanism for altering gene expression, and a number of genes encoding enzymes associated with histone modifications have been identified as tumor suppressors, for example, the histone deubiquitinase *BAP1* (Abdel-Rahman et al., 2011) and the histone demethylase *KDM6A* (Ler et al., 2017). Ubiquitination of the lysine at position 119 in histone 2A (H2AK119) blocks transcription by preventing RNA polymerase from traveling along the DNA template (Lanzuolo and Orlando, 2012). During transcriptional activation, H2A deubiquitinases remove ubiquitin from H2AK119 (Abdel-Wahab et al., 2012). A small number of these enzymes have been identified, among as many as 100 deubiquitinases in the human genome (Komander et al., 2009; Belle and Nijnik, 2014). These are six ubiquitin specific proteases (USPs; USP3, USP12, USP16, USP21, USP22, and USP46), one ubiquitin C-terminal hydrolase (BAP1) and one Zn^2+^ metalloprotease (MYSM1) (Chen et al., 2015). The present study focused on the role of the histone deubiquitinase USP16.

**FIGURE 1:**
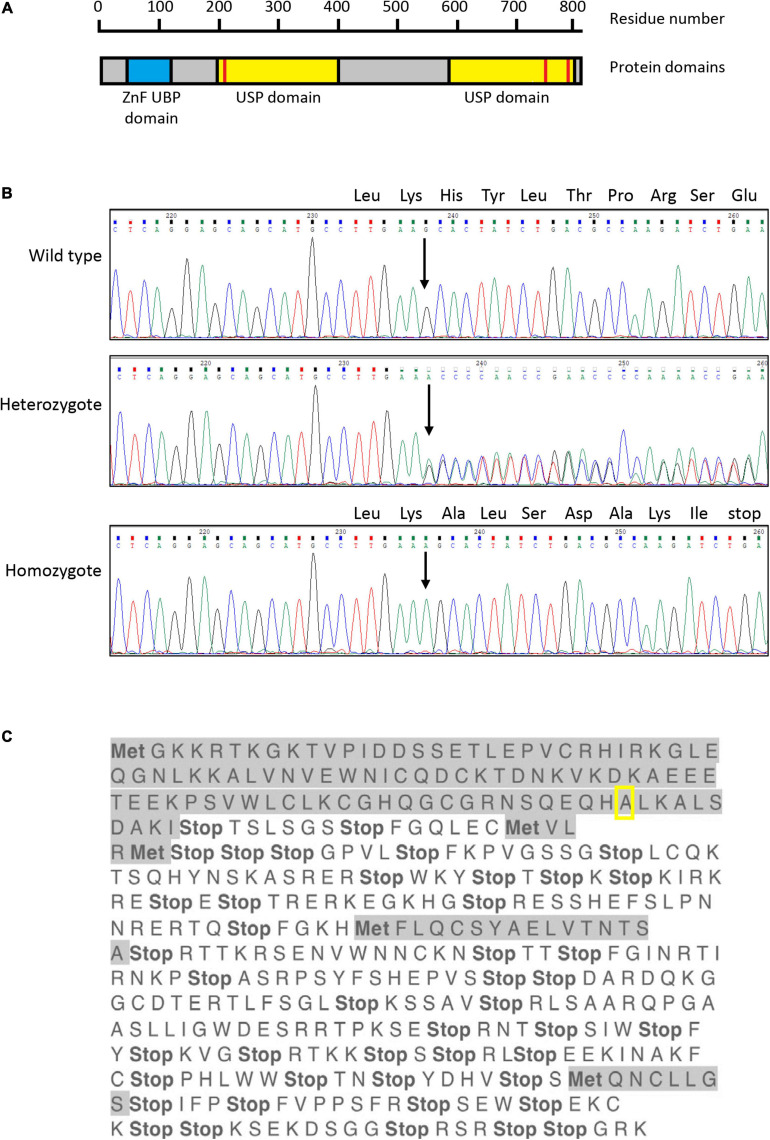
Sanger sequencing of exon 4 of *USP16* clones. **(A)** Structure of the human *USP16* gene and protein. Blue – ZnF UBP domain; yellow – ubiquitin specific protease (USP) domains; red bars – cysteine (left) and histidine (right) boxes of the C19 family peptidase domain. Asparagine at position 200 and cysteine at position 205 are key residues of the cysteine box; histidine at position 758 and aspartate at position 798 are key residues of the histidine box. The peptidase unit extends from residue 141 to the C terminus at residue 823. The USP domains are members of the C19 family of cysteine peptidases. Drawn from data in Jones et al. (2013), Rawlings and Barrett (2013) and the MEROPS database at https://www.ebi.ac.uk/merops/index.shtml (Rawlings et al., 2018). **(B)** Sequence of *USP16* in edited clones. Upper panel shows the wild type, middle panel shows a heterozygote and lower panel shows a homozygous edited clone. The site of the insertion is indicated by the arrow and the amino acid codons of the wild type allele are shown above the sequence traces. All three founder heterozygous clones had the same insertion of adenine (A; green trace) in one allele. The four homozygous *USP16*^–/–^ clones had the same insertion of one additional adenine, on both alleles. The nucleotide sequence before and after the insert was identical to wild type. (**C**) Translation of edited *USP16* in homozygous clones by the ExPaSy tool (single letter code). Insertion of the single nucleotide causes a frame shift, which introduces multiple stop codons in the translated sequence. The insert is present in the middle of the zinc finger domain, in codon 91 for the amino acid alanine (pictured here framed by yellow box). Ten codons later, translation would be terminated by an in frame stop codon. Met indicates potential start codons (amino acid methionine), and highlighted sequences show open reading frames. The first gray sequence up to the yellow box is the correct amino acid sequence.

USP16 (originally named UBP-M; Cai et al., 1999) is an 823 amino acid protein containing two domains; a zinc finger, ubiquitin-binding type domain (ZnF UBP domain; also called BUZ domain), which is also found in histone deacetylase 6 (HDAC6); and a catalytic site in the C19 family cysteine peptidase domain (Figure 1A). The ZnF UBP domain contains three zinc-binding sites consisting of 12 residues (Pai et al., 2007), which facilitate protein-protein and DNA-protein interactions, and the cysteine peptidase domain acts as a ubiquitin carboxyl-terminal hydrolase (Rawlings and Barrett, 1994). The protein was initially localized in cytoplasm (Cai et al., 1999). Subsequently it was found that USP16 functions as a homotetramer and is actively exported from the nucleus during interphase (Xu et al., 2013). At the onset of mitosis USP16 is phosphorylated at serine 552, by cyclin dependent kinase 1 (CDK1) (Xu et al., 2013) and localizes to the nucleus, where it is required for G2/M progression. The function of USP16 in mitosis is to deubiquitinate and therefore activate polo like kinase 1 (PLK1), which is needed for proper chromosome alignment, without which cell cycle progression is blocked at G2 (Joo et al., 2007).

USP16 can also act as a regulator of DNA damage repair. DNA double strand break damage induces H2A ubiquitination at the site of damage. The levels of *USP16* mRNA increase directly after DNA damage. After the break is repaired, the ubiquitin is removed by USP16 with the help of HERC2 (HECT and RLD domain containing E3 ubiquitin protein ligase 2) (Zhang et al., 2014). Over-expression of USP16 resulted in decreased ubiquitination of H2A immediately after the damage, while down-regulation resulted in increased and prolonged ubiquitination and failure to resolve the break (Zhang et al., 2014). The net result of either change was to reduce the cell’s ability to repair DNA damage, either because the initial ubiquitination of H2A at the site of the break was suppressed (over-expression of USP16) or because the ubiquitin could not then be removed after the repair (down-regulation) (Zhang et al., 2014). There are examples where *USP16* was down-regulated or mutated in leukemias and other human cancers, such as lung adenocarcinoma and hepatocellular carcinoma, which might contribute to the inability of cancer cells to resolve the DNA repair process (Fernandez et al., 2004; Gelsi-Boyer et al., 2008; Qian et al., 2016), but these are rare and the gene is ubiquitously expressed (data from FANTOM5 project^1^) and only rarely mutated (data from The Cancer Genome Atlas^2^).

USP16 may also contribute to inducible gene expression. USP16 was shown to be bound to promoter regions of various genes on all chromosomes of embryonic stem cells (ESC), and this was correlated with low H2A ubiquitination levels and high gene expression (Yang et al., 2014). USP16 was also identified to bind to active genes and promoters and take part in a shift of mouse B-cells from quiescent to active state (Frangini et al., 2013). In addition, USP16 regulates developmental *Hox* genes in *Xenopus laevis* (Joo et al., 2007).

Homozygous knockout of the *Usp16* gene (*Usp16*^–/–^) in mouse is embryonically lethal. Defects in development were found as early as 7.5 days after conception (E7.5) (Yang et al., 2014). USP16 catalytic function was required for ESC differentiation, but not ESC viability (Yang et al., 2014). Knock down of *USP16* in human HeLa cells caused slow cell growth rates, as there was a sharp decrease in cells in phase G2/M of the cell cycle (Joo et al., 2007). There was also a decrease in *HOX* gene expression. In *Usp16* conditional bone marrow knockout mice, the hematopoietic stem cells were reduced in maturity and lineage commitment and there were fewer mature cells in peripheral blood (Gu et al., 2016).

*USP16* is located on human chromosome 21, which is trisomic in Down syndrome (Adorno et al., 2013). In mice triplication of *Usp16* was associated with accelerated senescence, consistent with the early aging phenomena in patients with Down syndrome (Adorno et al., 2013). Consequences of overexpression of *Usp16* in mice included reduction of hematopoietic stem cells and their self-renewal ability, cellular defects owing to increased removal of H2A ubiquitin and decreased proliferation (Adorno et al., 2013). Patients with Down syndrome also have increased incidence of leukemia and decreased rate of solid tumors (Mateos et al., 2015). A human Down syndrome cell line with a triplicated *USP16* gene had decreased DNA damage response (Zhang et al., 2014).

These results show that USP16 has three functions: cell cycle progression (Xu et al., 2013), DNA damage repair (Zhang et al., 2014) and gene activation by removal of H2A ubiquitin (Yang et al., 2014). Disruption of each of these activities could contribute to generation and progression of leukemia.

Here we report the creation of a loss of function mutation in the *USP16* gene using CRISPR-Cas9 technology in the THP-1 human acute monocytic leukemia (AML) cell line. The THP-1 cell line was isolated in 1980 from a 1-year old boy suffering from AML. The cells resemble human monocytes (Tsuchiya et al., 1980) but can be induced to differentiate into macrophage-like cells by administration of phorbol myristate acetate (PMA) (Tsuchiya et al., 1982). PMA initially inhibits cell growth prior to differentiation, by up-regulation of the cyclin-dependent kinase CDKN1A; this in turn inhibits the activating phosphorylation of CDK2 (Traore et al., 2005). In the parent THP-1 line available from the American Type Culture Collection (ATCC^®^ TIB-202^TM^), around 50% of cells become adherent in response to PMA, indicating that there is considerable phenotypic heterogeneity (see Supplementary Figure 1 in Suzuki et al., 2009). To enable a detailed study of the transcriptomic response to PMA, the FANTOM consortium isolated a clonal line that was highly responsive to PMA and uniformly differentiated into a macrophage like state (Suzuki et al., 2009; Gazova et al., 2020). The karyotype of the clonal line was established by microarray-based comparative genomic hybridization to be 46XY, with minimal chromosomal aberrations (deletions at 6p, 12p, 17p, and on the X chromosome) (Adati et al., 2009). The THP-1 cells are also predisposed to mutations in tumor suppressor genes (for example *TP53* and *PTEN*) and *MLL* fusions (Adati et al., 2009).

We report the knockout of *USP16* in four homozygous cell lines derived from the high differentiation clone of THP-1. We observed heterogeneity amongst the homozygous knockout lines and examined their transcriptomic profiles to understand whether these cells have evolved mechanisms to compensate for the impact of the *USP16* mutation.

## Materials and Methods

### THP-1 Cell Line and Differentiation Assay

THP-1 cells (high differentiation clone 5, from FANTOM4 consortium, passage number 8, provided by Dr. Mark Barnett, The Roslin Institute, United Kingdom) were cultured as described previously (Gazova et al., 2020). The day before the start of the differentiation assay, cells were counted by hemocytometer and between 3 × 10^6^ and 5 × 10^6^ cells were pelleted and resuspended in 10 ml fresh medium. THP-1 cells were then differentiated by adding 30 ng/ml (48.6 nM) phorbol 12-myristate 13-acetate (PMA; P1585, Sigma-Aldrich) in DMSO. The cells were plated on a Sterilin plate (without tissue culture treatment of the plastic), and after 24 h of differentiation, the cells were lifted off by flushing with a blunt-end needle syringe.

### CRISPR-Cas9 Editing of *USP16* in THP-1 Cells

The CRISPR sequence guide was designed using an online website tool^3^, and selected based upon base chemistry and possible off-target effects. Information about human USP16 protein and gene structures was taken from the Ensembl database^4^ (Yates et al., 2016). Information was correct as of January 2021, based on transcript USP16-201 (ENST00000334352.8). The guide was designed to target exon 5 (5′-TGGCGTCAGATAGTGCTTCA-3′, score 79). *In silico* translation of the altered *USP16* DNA exonic sequence into protein was provided by the online ExPaSy tool^5^. Transcription start site (TSS) information was taken from the FANTOM5 database (Forrest et al., 2014) visualized on the ZENBU hg19 genome viewer^6^ (Severin et al., 2014). The oligonucleotides were ordered through Sigma-Aldrich (0.025 μmol, DST purification) (Ran et al., 2013).

Annealing and phosphorylation of the CRISPR guides followed the protocol of Andersson-Rolf et al. (2016). The phosphorylated and annealed oligonucleotides were diluted 1:200 and then cloned into a plasmid vector containing the *Streptococcus pyogenes* Cas9 open reading frame sequence and a GFP reporter gene (pX458 from Addgene, plasmid#48138, kindly provided by Dr. Peter Hohenstein, The Roslin Institute) in one step. Cutting and ligation was done by adding together 100 ng of pX458, 2 μl of the diluted annealed oligonucleotide, 2 μl of 10X T4 ligase buffer with 10mM ATP (NEB, Ipswich, MA, United States), 1 μl of *Bbs*I restriction endonuclease (10 U/μl, NEB), 0.5 μl of ligase from Quick Ligation kit (NEB) made up to 20 μl with water. The mixture was incubated for 6 cycles of 37°C for 5 min and 21°C for 5 min. The plasmid with sgRNA sequence was treated using the Plasmid Safe Exonuclease kit (Epicentre, Madison, WI, United States) which digests any residual linear DNA, following manufacturer’s instructions.

DH5α strain *E. coli* bacteria were then transformed with the plasmid using a heat shock at 42°C and the bacteria were streaked on an ampicillin plate (100 μg/ml ampicillin in LB (Lysogeny Broth) agar). Two colonies per plate were selected and grown overnight in 5 ml LB with 100 μg/ml ampicillin. To assess the presence of the appropriate insert, plasmid DNA was extracted using the Qiagen MiniPrep kit according to manufacturer’s instructions (Qiagen, Hilden, Germany). The sequence was validated by chain termination sequencing using U6 FWD primer at Edinburgh Genomics (University of Edinburgh, United Kingdom). A large scale preparation of plasmid DNA was then made using the Endo-free Maxi Prep (Qiagen) according to manufacturer’s instructions. It was important to use endotoxin-free (Endo-free) reagents to avoid activating the cells during the transfection step. The sequence of the maxi prep-prepared plasmid was again verified by sequencing at Edinburgh Genomics.

### Nucleofection of CRISPR-Cas9 Plasmids Into THP-1 Cells

THP-1 cells were transfected using the 4D Nucleofector kit (Lonza, Cologne, Germany), with a Lonza protocol optimized for THP-1 cells. 1 × 10^6^ THP-1 cells per sample were centrifuged at 400 *g* for 5 mins, the pellet resuspended in 100 μl of SG 4D Nucleofector solution with added supplement (SG Cell line 4D Nucleofector solution X kit, Lonza). 0.5 μg of DNA per sample of Endotoxin-free plasmid was added. The cell suspension was transferred to the Nucleocuvette vessels and the program FF-100 was executed on the 4D Nucleofector. 500 μl of pre-warmed THP-1 medium was then added and the cell suspension was transfered to a 12-well plate with 1 ml of THP-1 medium already in each well. Next day (∼24 h after nucleofection), the cells were spun down at 400 g for 5 min at room temperature, and the pellet resuspended in 300 μl of 10% fetal bovine serum in PBS and subjected to flow cytometry assisted cell sorting. Single GFP positive cells (which had taken up the plasmid) were sorted into 96-well plates with 200 μl THP-1 media per well, using a BD FACS Aria IIIu (BD Biosciences, San Jose, CA, United States).

### Validation of Knockout Cell Lines

The single cell clones were left to grow in 96 well plates until there were enough cells to passage into a bigger vessel (multiple weeks). DNA was prepared from these potential knockout clones using phenol extraction. DNA was resuspended using a suitable volume of TE or water. The targeted region was then amplified by the polymerase chain reaction using High Fidelity Q5 Polymerase (NEB) according to manufacturer’s instructions. The primer sequences were CCTAGCGAGTGCATGGTTTT (USP16 CRISPR site F) and ACCCAAGAGGCAGAGGAACT (USP16 CRISPR site R) and the T_m_ for both was 65°C (NEB Tm Calculator^7^). The samples were initially denatured at 98°C for 30 s, then incubated for 35 cycles of 98°C for 10 s, 65°C for 30 s and 72°C for 30 s. The final extension was at 72°C for 2 mins. The PCR product was run on a 1.5% agarose gel (Agarose Ultrapure, Invitrogen, Paisley, United Kingdom) in 1X TAE with 1X of SYBR Safe DNA stain (Invitrogen). 20 μl of the PCR product was purified using Charge Switch PCR clean-up kit (Invitrogen) according to manufacturer’s instructions. For sequencing, 3 μl of water, 2 μl of the purified PCR product and 1 μl of 3.2 μM primer was mixed and sent for chain termination (Sanger) sequencing at Edinburgh Genomics. The results were viewed using FinchTV programme (Geospiza, Perkin Elmer, Waltham, MA, United States) or Chromas^8^.

### Gene Expression Analysis

RNA was extracted from pelleted cells using RNABee (AMS Biosciences, Friendswood, TX, United States) or TRIzol (Thermo Fisher Scientific, Waltham, MA, United States) according to the manufacturers’ instructions. The RNA samples were then treated with DNase I according to manufacturer’s instructions (Ambion DNase kit AM1906, Thermo Fisher Scientific). The concentration of RNA was measured using a NanoDrop spectrophotometer ND-1000 (Nanodrop Technologies, Wilmington, DE, United States) and quality was assessed using the Agilent RNA ScreenTape System (Agilent Technologies, Santa Clara, CA, United States) according to the manufacturer’s instructions. 500 ng DNase I treated RNA was used to prepare cDNA, together with 2 μl random primers (50 ng/μl, Invitrogen) and 1 μl dNTPs (10 mM, Invitrogen), with water to 13 μl. The RNA with random primers was denatured at 65°C for 5 mins and then cooled at 4°C for at least 1 min. Afterward, 4 μl 5x first strand buffer (Invitrogen), 1 μl 0.1 M DTT (Invitrogen), 1 μl RNAsin Plus (Promega, Madison, WI, United States) and 1 μl Superscript III Reverse Transcriptase (RT; Invitrogen) were added to the mixture. An RT negative control was prepared using RNAse-free water instead of RT. The cDNA synthesis reaction was incubated at 25°C for 5 mins, 50°C for 60 mins, and finally 70°C for 15 mins to stop the reaction. Before quantitative polymerase chain reaction (qPCR), the cDNA was diluted 1:1 with 20 μl water. To establish standard curves, cDNA from THP-1 RNA was diluted three times. The first point of the standard curve was undiluted cDNA (estimated 12.5 ng/μl), then 1:1 (estimated 6.25 ng/μl), then 1:4 (estimated 3.125 ng/μl), and 1:8 (estimated 1.5625 ng/μl). qPCR was used to assess the levels of *USP16* expression using manufacturer’s protocols for SYBR Green 1 Master Mix with Light Cycler 480 96-well white plates (Roche, Mannheim, Germany). The settings for all qPCR analyses (both quantification and melting curves for primers) were as follows: pre-incubation was carried out at 95°C for 5 mins (ramp rate 4.40°C/s), then amplification steps were repeated for 45 cycles. Amplification steps were as follows: 95°C for 10 s (ramp rate 4.40°C/s), 60°C for 15 s (ramp rate 2.20°C/s), and 70°C for 30 s (ramp rate 4.40°C/s). Afterward, the melting curve was measured by incubating at 95°C for 5 s (ramp rate 4.40°C/s), 65°C for 1 min (ramp rate 2.20°C/s) and then the temperature was increased to 97°C by 0.11C°/s. At the end, the plate was cooled for 30 s at 40°C. All reverse transcriptase-qPCR analysis was carried out using the Advanced Quantification setting of the Light Cycler 480 Roche software. ΔCt was calculated with previously established values of primer efficiencies from standard curves (calculated using the same software, by using Abs Quant/2nd Derivative Max setting).

Two sets of primers for two different housekeeping genes were used in this study. The first one was for the human beta actin gene (*ACTB*) (Maess et al., 2010); the second, for *GAPDH*, was purchased from Qiagen (QuantiTect Primer Assay QT0112646). The *USP16* primers were designed to span an intron, to have melting temperature (Tm) of 60°C and to generate a cDNA product of approximately 200 bp, using Primer3 program^9^. The ideal slope value from standard curves is around −3.345 when the primer efficiency is 2, but values from −3.0 to −3.5 were considered acceptable (Table 1).

**TABLE 1:** qPCR primers and efficiencies for *USP16* expression analysis.

Gene target	Sequence 5′ – 3′	Slope value
USP16_ex4-5_F	TGCCAAGACTGTAAGACTGACA	−3.543
USP16_ex4-5_R	TGGCGTCAGATAGTGCTTCA	
USP16_ex15-16_F	AGTATGCACACGGAGACAGT	−3.528
USP16_ex15-16_R	AGAGTAAGAACAGGAGGAGCA	
USP16_ex17-18_F	CCTACGCAAAGTTAACAAACACA	−3.014
USP16_ex17-18_R	GTGTAATGCCCCGACCTCAT	
ACTB_F	ATTGCCGACAGGATGCAGAA	−3.398
ACTB_R	GCTGATCCACATCTGCTGGAA	
GAPDH	Qiagen	−3.498

### CAGE (Cap Analysis Gene Expression)

Cap analysis gene expression libraries were made as described previously (Takahashi et al., 2012; Gazova et al., 2020). Libraries were sequenced by Edinburgh Genomics on an Illumina HiSeq 2500 machine in high throughput mode. One library consisting of eight pooled samples was sequenced per lane, with custom sequencing primer and inline barcodes. Quality control, quantification of expression levels and bioinformatic analysis were performed as described (Gazova et al., 2020). The final expression value for each CTSS (CAGE transcription start site) was provided as TPM (tags per million). The normalized data were then formatted into OSCtable^10^, providing chromosome, start, end and strand coordinates and uploaded into ZENBU^11^ (Severin et al., 2014). CTSS were clustered in each sample based on their distance apart using distclu option in CAGEr package (Haberle et al., 2015). The settings were as follows, the minimum CTSS TPM value was 1, the distance between CTSS was maximum of 20 bp and singletons (single CTSS not neighboring any other CTSS) were not removed. To compare the clustered CTSS across different samples, the CTSS range values were aggregated, retaining only the CTSS clusters with expression in at least one sample of higher than 5 TPM. The maximum distance between CTSS was kept at 100 bp. These commands created a single matrix file with cluster coordinates (start, end, strand) and normalized TPM values for the aggregated clusters. Normalized and annotated clusters of CTSS were allocated to the nearest downstream gene (Gazova et al., 2020). A matrix of gene annotations and expression values was uploaded into BioLayout software (Freeman et al., 2007; Theocharidis et al., 2009)^12^. Sample-to-sample correlation (equivalent to principal components analysis) was created by transposing the data in pre-processing and analyzed at a Pearson correlation coefficient (*r*) threshold of 0.88. Gene network analysis created a graph by visualizing each node as one aggregated cluster of CTSS. A correlation graph was created at *r* ≥ 0.92 (Supplementary Figure 1) using Fast Multiple Multilevel Method (FMMM) format. The analysis was done by clustering using the Markov Cluster Algorithm (MCL) at inflation values indicated in the Results section. Differential gene expression was analyzed using the edge package for the R statistical environment^13^. GO enrichment analysis was performed with PANTHER (Protein Analysis Through Evolutionary Relationships, release of July 28, 2020; available through The Gene Ontology Resource^14^). The background was *Homo sapiens*, the test used was Fisher’s Exact test and all results were corrected for multiple testing using the Bonferroni method.

### Western Blot

For each sample, 2 × 10^6^ cells were resuspended in 50 μl PBS, then 50 μl of 2X Laemmli loading buffer with 50 mM DTT (BioRAD, Hercules, CA, United States, prepared by mixing 950 μl Laemmli loading buffer + 50 μl 1M DTT (NEB) in water) was added and mixed thoroughly. Samples were then incubated at 95°C for 5 mins and stored at −20°C until needed. Samples were loaded on a precast Mini-Protean TGX 4-15% 12-well gel (Bio-Rad Laboratories, Herclues, CA, United States). A molecular weight marker (PageRuler Plus Prestained Protein Ladder, Thermo Fisher Scientific) was loaded in the outer lanes. The running buffer was 25 mM Tris (Thermo Fisher Scientific), 192 mM Glycine (Sigma-Aldrich) and 0.1% w/v SDS (Thermo Fisher Scientific) and the gel was run at 100V for 5 min and then 120V till the end of the gel. The gel was then rinsed in water. The protein was then transferred onto PVDF membrane (Immobilon-P, Sigma-Aldrich, St Louis, MO, United States), using a Bio-Rad transfer apparatus, according to manufacturers’ instructions. The blotting was run at 50 V for 1 h at initial current of 400 mA. The membrane was blocked for 1 h in 5% milk powder (Marvel Dried Milk, Premier Foods Group Ltd., London, United Kingdom) in PBS-T (0.05% Tween in PBS). Primary anti-human antibodies (dilution of 1:1000 for rabbit anti-USP16 (ab121650, Abcam, Cambridge, United Kingdom); and 1:2000 dilution for mouse anti-ß-actin (C4) monoclonal IgG1 (sc-47778, Santa Cruz Biotech, Dallas, TX, United States) were diluted in 5% milk powder in PBS-T and the membrane immersed rotating overnight at room temperature. The next day, the membrane was washed six times for 5 min in PBS-T, and then secondary antibodies were diluted at 1:2000 in 5% milk powder in PBS-T (horse anti-mouse HRP-linked for β-actin and goat anti-rabbit HPR-linked for USP16; both from Cell Signaling Technology, Danvers, MA, United States), and the membrane immersed rotating for 1 h at room temperature. The membrane was again washed six times for 5 min in PBS-T, and then Pierce ECL western blotting substrate (Thermo Fisher Scientific) was applied according to manufacturer’s instructions. The image was developed onto Amersham Hyperfilm (GE Healthcare, Little Chalfont, United Kingdom).

### Analysis of Edited Cells

MTT assay was performed to assess metabolic activity and cell proliferation. A 96-well plate was seeded with 2 × 10^4^ THP-1 cells in 100 μl of media per well. After 48 h, 10 μl MTT (3-(4,5-dimethylthiazol-2-yl)-2,5-diphenyltetrazolium bromide, 5 mg/ml, Sigma Aldrich) was added to each well and incubated for 3 h at 37°C in 5% CO_2_. Then 100 μl solubilization solution (89% (v/v) Isopropanol, 10% (v/v) Triton 100x, 1% (v/v) HCl) was added and left at 37°C 5% CO_2_ overnight. The following day, substrate conversion was determined via the optical densities which were measured using a plate reader at 570 nm. The phases of the cell cycle were determined using propidium iodide staining, as described previously (Gazova et al., 2020).

A phagocytosis assay was performed by incubating THP-1 macrophages that had been differentiated with PMA for 2 days with Zymosan A particles (Thermo Fisher Scientific, Z2841) coated with fluorescein isothiocyanate (FITC), at a ratio of 100 particles per cell; for 1 h at 37°C. Cells were washed five times with cold PBS and fixed with 4% paraformaldehyde (PFA, WVR, Radnor, PA, United States) for 10 min at room temperature and washed twice with PBS. Images were viewed using a fluorescent microscope (Zeiss Vert.A.1, Carl Zeiss Limited, Cambridge, United Kingdom).

### Statistical Analysis

Results are presented as mean ± standard error. Significance of differences was assessed using a two tailed *t*-test where two groups were compared or a two tailed *Z*-test where a single sample was compared with the mean of multiple values.

## Results

### Editing of the *USP16* Gene in THP-1 Cells

The guide was designed to make an indel in exon 5 of *USP16*, creating an out of frame mutation. Exon 5 (the third coding exon) codes for part of the zinc finger domain. Out of two 96 well plates, 15 clones survived the sorting and 3 clones proved to be heterozygous for the same single nucleotide insertion (adenine) into the expected site. The sequence is shown in Figure 1B. No homozygotes were present at the first targeting, so two of the heterozygotes were used for targeting a second time with the same guide. Out of 3 plates for each heterozygous parental cell line, 67 clones survived the sorting. 13 clones (19%) had reverted to the wild type sequence and 4 clones (6%) were homozygous for the same adenine insertion in exon 5 (Figure 1B). The insertion site of the adenine in exon 5 created a frame shift, with an in-frame stop codon ten amino acids downstream. Any resulting peptide would be small and contain only the start of the protein sequence with no functional domains (Figure 1C). One homozygous clone (Hom1) was derived from one heterozygote (HetC) and the other three (Hom2, Hom3, and Hom4) from a second heterozygote. Sequencing of the cDNA derived from the homozygous clones confirmed that the insertion was present in the transcript as well as the gene (not shown).

### Impact of *USP16* Frame Shift on Gene Expression

**FIGURE 2:**
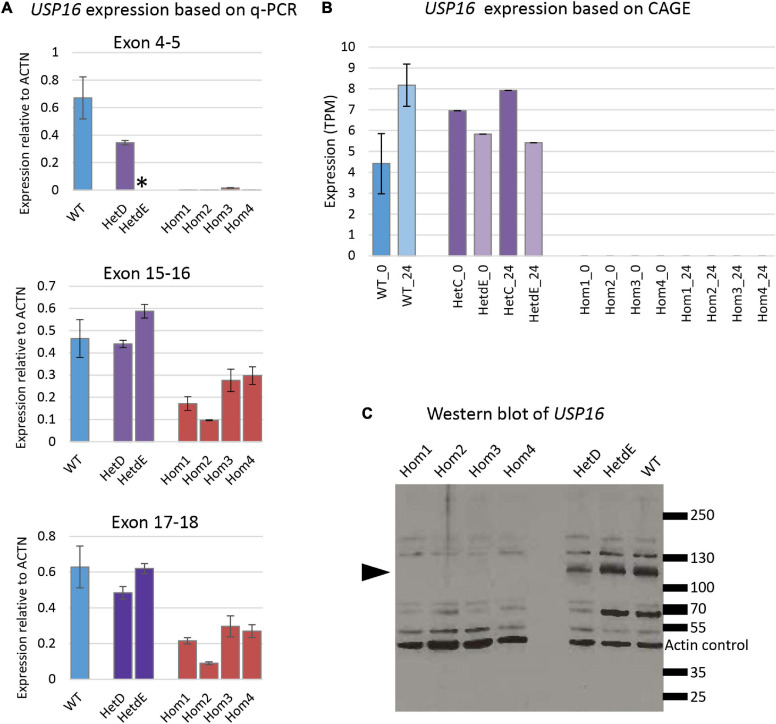
*USP16* expression in edited clones. **(A)** Quantitative PCR for three sets of *USP16* primers, targeting different exons. RNA was from undifferentiated cells. Blue – wild type; purple – heterozygotes; red – homozygotes. *Y*-axis shows the Roche ratio normalized by *ACTB* expression with error rates calculated by Roche LightCycler480 software. Error bars show standard error. Asterisk shows technical failure of qPCR for HetdE. All assays were carried out in duplicate. Homozygotes were significantly different from wild type (all *p* < 10^–7^); heterozygotes were not significantly different from wild type. **(B)** *USP16* expression derived from transcriptomic analysis using CAGE. Expression levels are shown for control untreated cells (darker columns) and cells after 24 h of PMA stimulation (lighter columns). Blue – wild type; purple – heterozygotes; values in homozygotes were all 0. Homozygotes were significantly different from wild type (*p* = 0.03 at 0 h and *p* = 0.004 at 24 h). Heterozygotes were not significantly different from wild type at either time point. **(C)** Western blotting for USP16 and actin in extracts of THP-1 clones. Lanes 1, 2, 3, and 4 are *USP16* knockout homozygotes; lanes 6 and 7 are *USP16* knockout heterozygotes, and lane 8 shows the wild type THP-1 cell line. Molecular weight ladder sizes (kDa) are shown to the right of the image. Actin loading control bands of 42 kDa are indicated. All other bands were detected by anti-USP16 antibody (ab121650). Bands of around 120 kDa (present in heterozygotes and wild type) were not present in the four homozygous *USP16* knockout THP-1 clones (arrowhead).

Quantitative reverse transcriptase PCR experiments were conducted with the four homozygotes, a parental heterozygote (HetC), a heterozygote from the second targeting (HetdE) and wild type THP-1 cells for 3 sets of primers, each amplifying a different exon along the transcript (Table 1). Consistent with predicted nonsense-mediated decay, the *USP16 ^–/–^* homozygous clones had decreased level of mRNA compared to wild type THP-1 cells (Figure 2A) for all three sets of primers (*p* < 10^–7^ for each set of primers). The *USP16* expression levels were also extracted from the transcriptomic data generated by CAGE analysis (see below) and found to be negligible in the homozygous edited clones after differentiation (*p* = 0.004) (Figure 2B).

The production of USP16 protein in the edited clones was assessed by western blotting. Previous studies reported an estimated molecular weight for USP16 of 110 - 120 kDa (Joo et al., 2007; Xu et al., 2013), although the antibody manufacturer predicted a band size of 94 kDa. A band of around 120 kDa was present in heterozygotes and wild type but absent in the homozygous clones (Figure 2C). There was no evidence of any bands between 70 and 130 kDa in the homozygotes. This suggests that the homozygous clones were not making full length USP16. Other bands on the gel (Figure 2C) that were not altered in the deleted clones may be from different deubiquitinases, since there is strong homology in the family.

### Cellular Impact in *USP16* Knockout Cells

**FIGURE 3:**
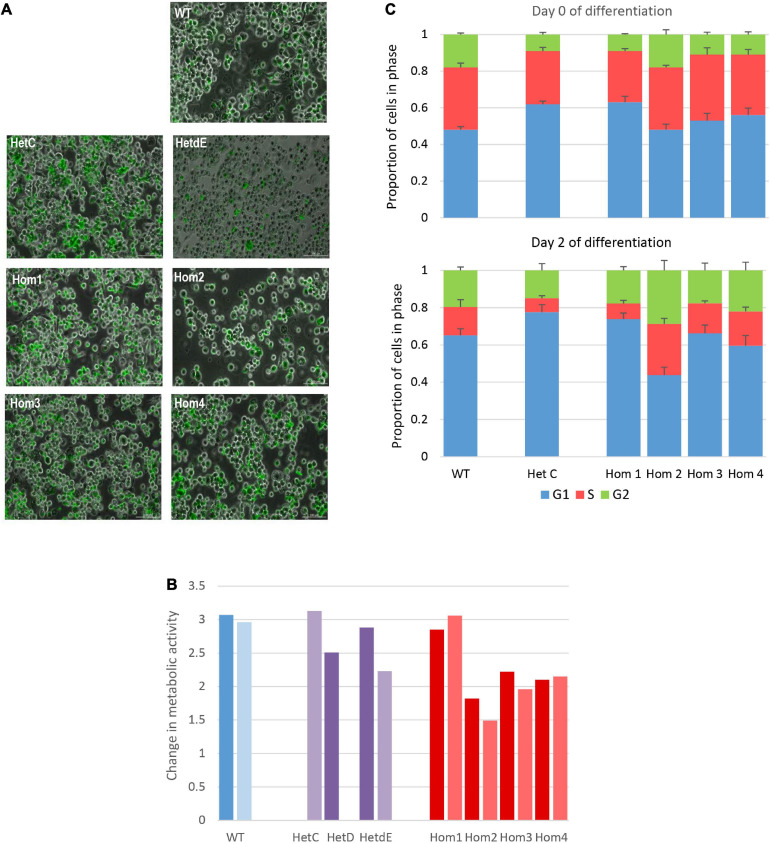
Impact of *USP16* knockout on cellular functions. **(A)** Phagocytosis of Zymosan particles by wild type and edited clones. Adherent THP-1 cells differentiated with PMA were incubated with FITC-labeled Zymosan particles for 1 h at 37°C and washed as described in Methods. Images show that the large majority of cells in each culture contained labeled (green) particles. **(B)** Proliferation of individual THP-1 clones measured using the MTT reduction assay. Cells were plated at 2 × 10^5^/ml and incubated for 48 h, prior to assay of viable cells as described in Methods section. Dark colors show an assay after initial expansion; light colors show a repeat assay of the same clones, after several passages. *Y*-axis shows the optical density after 48 h. Red – homozygotes; purple – heterozygotes; blue – wild type. HetD was only tested after the initial expansion and HetC was only tested after several passages. **(C)** Proportion of cells in different stages of the cell cycle in wild type and edited clones. Cell cycle was analyzed by flow cytometry of propidium iodide-stained cells as described in Methods. Results are the average of 3 (HetC) or 4 experiments. Upper panel – before differentiation; lower panel – after 2 days with PMA. Blue – G1 phase; orange – S phase; green – G2 phase. Error bars show standard error.

The *USP16* knockout clones retained active proliferation in the absence of PMA, indicating that USP16 is not absolutely required for mitosis in these leukemic cells. Upon addition of PMA the parent THP-1 *USP16*^+/+^ high differentiation clone underwent growth arrest, with down-regulation of cell-cycle associated transcripts and *MYB*, and up-regulation of macrophage markers (Gazova et al., 2020). The absence of functional USP16 did not alter the ability of the cells to undergo this differentiation process. In control and USP16-deficient cells, addition of PMA generated a confluent layer of adherent macrophage-like cells after 48 h. These cells were able to phagocytose Zymosan A particles and there was no apparent difference between the control and USP-deficient clones (Figure 3A).

To analyze the requirement for USP16 in growth regulation of these cells, we first assessed proliferation based upon metabolic activity (MTT reduction). This was assessed on both early and late passage lines to determine whether there was any phenotypic drift. The results differed among *USP16*^–/–^ homozygotes. The proliferative activity of wild type, heterozygotes and homozygote Hom1 was indistinguishable, as indicated by the level of metabolic activity after 48 h in culture. The remaining homozygotes (Hom2, Hom3, and Hom4, all derived from the same heterozygote) were also actively proliferative albeit with a marginal decrease in MTT reduction compared to WT (Figure 3B).

To assess the impact of *USP16* mutation on cell cycle regulation, the various clones were differentiated into macrophages with PMA over the course of 3 days and the proportions of cells in different phases of the cell cycle were assayed each day using propidium iodide staining and flow cytometry. This analysis confirmed that USP16 is not absolutely required for cell cycle progression. Before differentiation, edited and wild type cells were indistinguishable, with 20-30% of cells in S phase (Figure 3C). During differentiation of THP-1 *USP16*^+/+^ monocytes to macrophages in response to PMA the proportion of cells in S phase was previously shown to decline as the cells differentiate (Gazova et al., 2020). This response was replicated here in wild type and in *USP16* heterozygous and homozygous cells, although the decline in Hom2 was not as marked. The proportion of cells in S phase was reduced to half or less in most lines alongside an increased proportion in G1 phase by Day 2. In Hom2, the proportion of cells in S phase following PMA treatment was maintained around 30% (Figure 3C). As indicated by the error bars in Figure 3C, there was considerable variability between replicates.

### Transcriptomic Analysis of Wild Type THP-1 and *USP16* Knockout Clones

**FIGURE 4:**
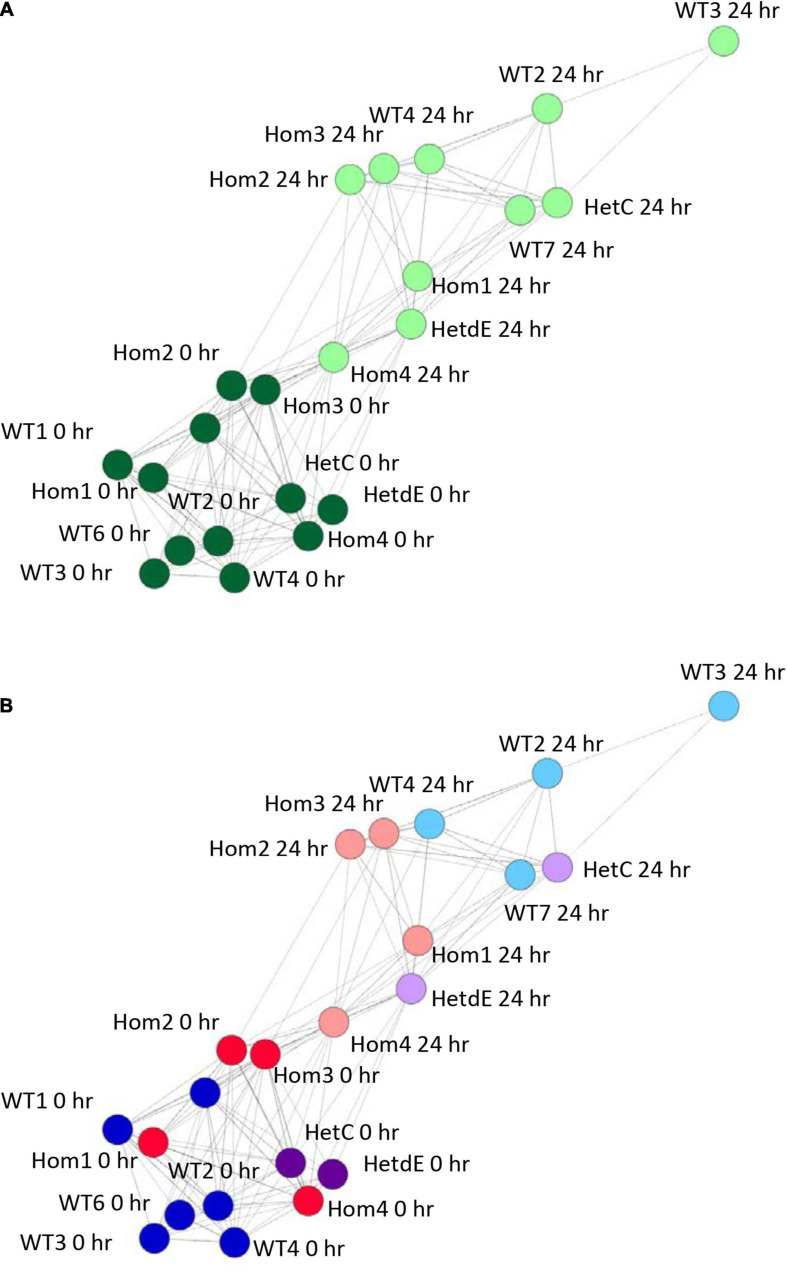
Sample to sample network graph of edited clones. Circles represent clones and lines between them correlations of at least 0.88. Wild type results are from Gazova et al. (2020). **(A)** Analysis showing the transition from 0 h (dark green) to 24 h post PMA treatment (light green). **(B)** The same network as in panel **(A)**, colored to show the effect of genotype on the transition following PMA treatment. Dark colors show cells prior to treatment, light colors show cells 24 h after PMA treatment. Red – homozygotes; purple – heterozygotes; blue – wild type.

The phenotypic analysis indicated that the impact of the *USP16* insertion is conditional; in one clone (Hom2) there was a reduced impact on proliferation though not on differentiation, whereas Hom1 was not different from its heterozygous parent HetC or the replicates from the original wild type THP-1 clone. To dissect the reasons for this variable impact, RNA from *USP16* knockout THP-1 cells was subjected to expression analysis using CAGE before and after 24 h PMA stimulation, and compared with results for wild type using CAGE sequencing results from the 0 and 24 h time points of the previous publication (Gazova et al., 2020). Sample-to-sample analysis (which provides similar information to a principal components analysis) using BioLayout network analysis software showed that prior to PMA treatment, all cells had similar transcriptomic profiles (dark green in Figure 4A). All cell lines showed some change over the first 24 h of differentiation, with the transcriptome of the wild type cells changing most and Hom4 changing least (Figure 4B). One heterozygote (HetC) was similar to wild type and the other (HetdE, a heterozygous clone that had been through two rounds of targeting, from the same parent as Hom2, Hom3, and Hom4) was similar to the homozygotes, which were closest to undifferentiated cells in the network, indicating a reduced response to PMA (Figure 4B).

**FIGURE 5:**
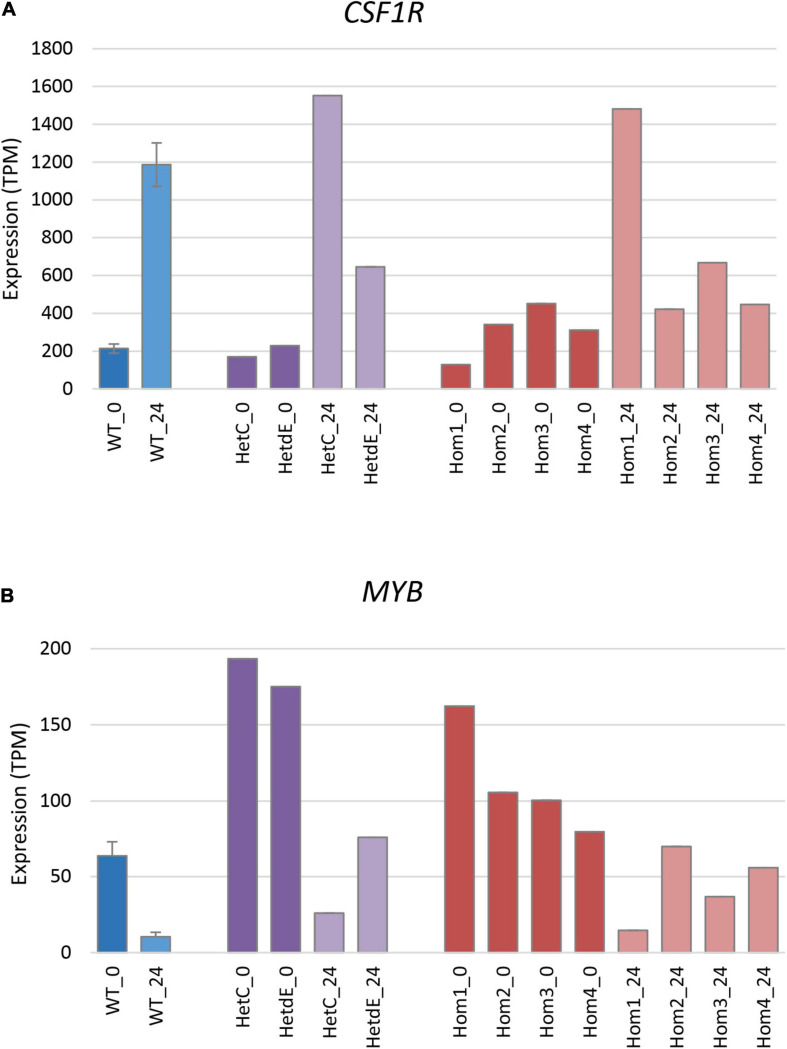
Expression of signature genes in edited clones. Results are based on expression levels extracted from CAGE data. Wild type samples are from Gazova et al. (2020); results are the mean of 6 (0 time point) and 4 (24 h time point) replicates and standard error is shown by the bars. (**A**) Expression of *CSF1R* (total of three promoters), a marker of monocyte to macrophage transition. At 0 h, Hom1 was significantly lower than wild type (*p* = 0.0006) while at 24 h it was significantly higher (*p* = 0.01). The mean value for Hom2-4 was slightly higher than wild type at 0 h (*p* = 0.04) but did not change after PMA stimulation and was then significantly lower than wild type (*p* = 0.005). (**B**) Expression of *MYB* (total of two promoters), a marker of cell proliferation. At 0 h, Hom1 was significantly higher than wild type (*p* = 0) while at 24 h it was not significantly different (*p* = 0.15). The mean value for Hom2-4 was slightly higher than wild type at both 0 and 24 h (*p* = 0.04 in both cases).

The differentiation of THP-1 cells is associated with up-regulation of a number of macrophage-specific genes, including *CSF1R*, which encodes the receptor for the lineage-specific growth factors CSF1 and IL34 (Hume et al., 2016; Gazova et al., 2020). The three related homozygous knockout lines (Hom2, Hom3, and Hom4) showed no increase of *CSF1R* mRNA after PMA treatment (*p* = 0.19; Figure 5A), while their double targeted “sibling” HetdE showed a small (3-fold) increase. In contrast, Hom1 and its parental heterozygote HetC showed approximately 10-fold increase in *CSF1R*, greater than the 5-fold increase seen in wild type. The progenitor cell marker *MYB* decreased to about 15% in wild type after PMA treatment. This marker was higher in Hom1 and HetC than in wild type prior to treatment and decreased to less than 15% 1 day after PMA treatment. In the double targeted HetdE, Myb expression prior to PMA treatment was similar to HetC, but only decreased to about 40%. In Hom2, Hom3 and Hom4, *MYB* expression was lower prior to differentiation and dropped up to 50% after PMA treatment (*p* = 0.03) indicating that cell proliferation persisted in these clones (Figure 5B).

**FIGURE 6:**
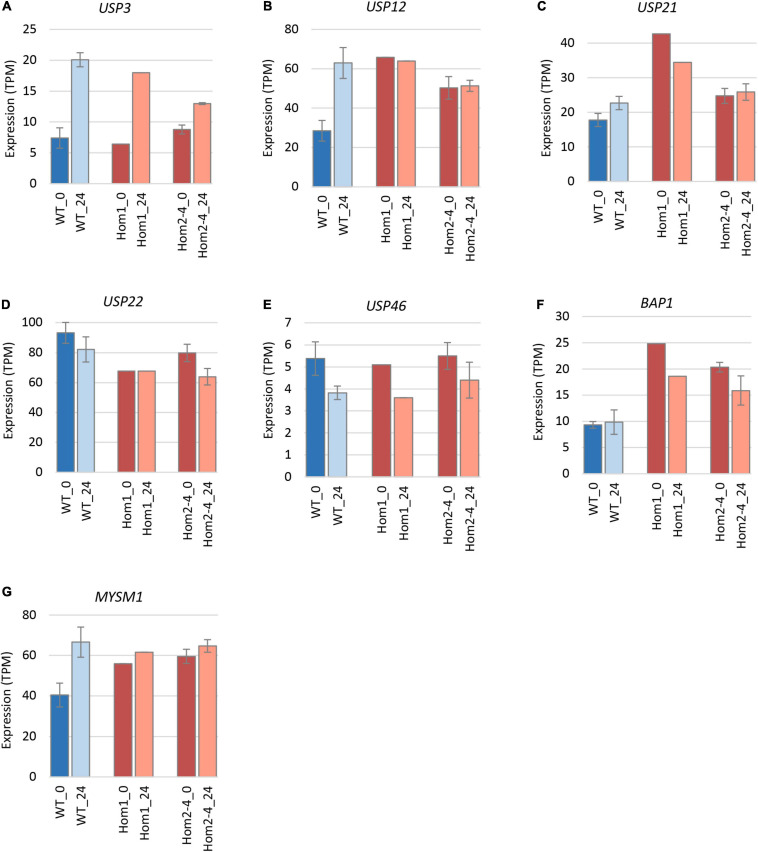
Expression of deubiquitinase genes in edited clones. Results are based on expression levels extracted from CAGE data. Wild type samples are from Gazova et al. (2020). Results for wild type are the mean of 6 (0 h time point) and 4 (24 h time point) replicates. Results for Hom2, Hom3, and Hom4 were averaged. Standard error is shown by the bars. Significance of differences between genotypes can be seen in Table 2. **(A)** Expression of *USP3*. **(B)** Expression of *USP12*. **(C)** Expression of *USP21*. **(D)** Expression of *USP22*. **(E)** Expression of *USP46*. **(F)** Expression of *BAP1*. **(G)** Expression of *MYSM1*.

To explain the apparent redundancy of USP16 for cell proliferation and differentiation, and inconsistency between the mutant clones, we examined expression of other histone deubiquitinase genes. Because Hom1 was different from the other homozygotes, it was analyzed separately. All homozygotes had higher levels of expression prior to differentiation for *USP12, BAP1*, and *MYSM1* (Figure 6 and Table 2). *USP12* and *MYSM1* were induced in wild type cells upon PMA treatment, reaching the same level as the homozygotes. *BAP1* did not change following PMA treatment and was significantly elevated in Hom1 regardless of time point. Hom1 was also higher than wild type for *USP21* both before and after treatment but lower than wild type for *USP22* prior to treatment (Figure 6 and Table 2). Expression of *USP3* in Hom2, Hom3, and Hom4 was lower than wild type after 24 h of PMA treatment, suggesting that these clones failed to fully induce *USP3* during differentiation. There was no effect of the *USP16* knockout on *USP46*, which had relatively low expression in these cells.

**TABLE 2:** Expression of deubiquitinases in USP16 edited cells.

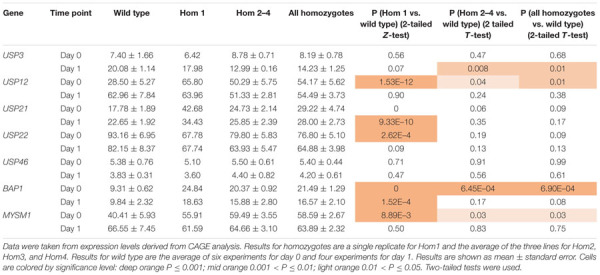

### Network Analysis of *USP16* Edited Clones

**FIGURE 7:**
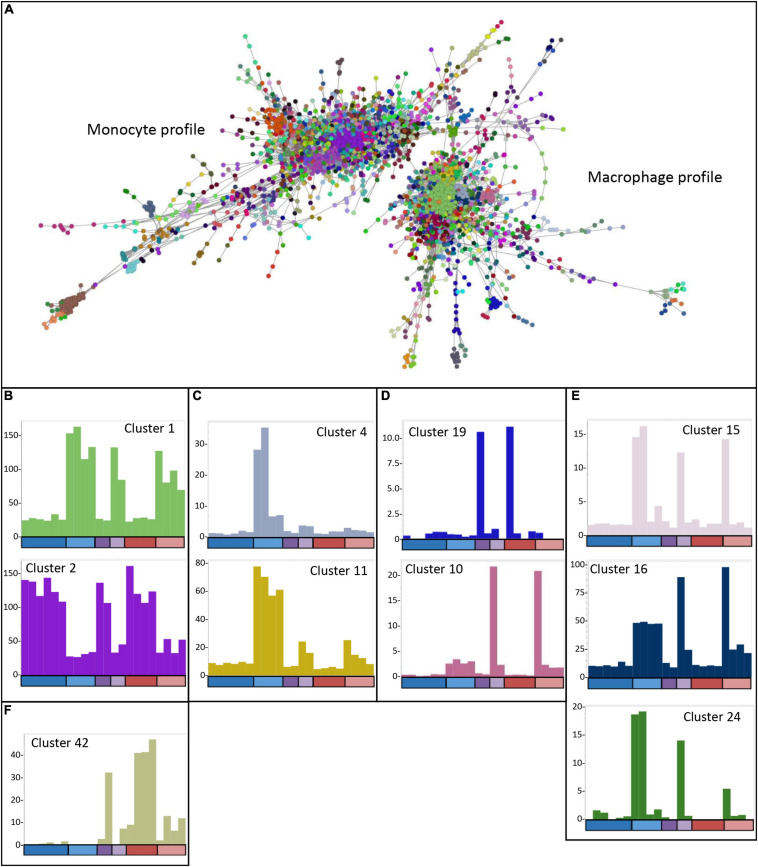
Network analysis of edited cells. **(A)** Gene-to-gene 2D network graph. Image taken from BioLayout output showing CTSS-CTSS correlations (nodes = CTSS; edges = correlations of ≥0.92 between them). The analysis included all four *USP16* knockout lines, HetC and HetdE and wild type samples; raw data for wild type are from (Gazova et al., 2020), 0 h and 24 h time points. Nodes in the same cluster have the same color. **(B)** Average expression profiles for Clusters 1 and 2, showing up- or down-regulation in response to PMA in all cells. **(C)** Average expression profiles for Clusters 4 and 11 showing very small effect of PMA in the edited homozygous and heterozygous cells. **(D)** Average expression profiles for Clusters 10 and 19, showing specific expression in Hom1 and its parent HetC at 0 h (cluster 19) or 24 h post PMA treatment (Cluster 10). **(E)** Average expression profiles for Clusters 15, 16, and 24 showing expression in some or all wild type samples as well as HetC and Hom1. **(F)** Average expression profile for Cluster 42, showing high expression only in HetdE, Hom2, Hom3, and Hom4. In panels (**B**-**F)**, *Y*-axis shows the average expression of genes in the cluster in TPM. *X*-axis shows the samples. The bar along the X axis shows the time point, colored as for Figure 5: dark colors 0 h, light colors – 24 h after PMA stimulation; blue – wild type, purple – heterozygotes, red – homozygotes. Samples are in the order wild type (6 samples at 0 h and 4 at 24 h), heterozygotes (HetC, HetdE), homozygotes (Hom1, Hom2, Hom3, and Hom4). Columns have the same color as the clustered nodes in panel **(A)**.

The analysis of differentially expressed genes and determination of the phenotypes of the edited clones indicated that there were impacts of the lack of USP16 in both homozygotes and heterozygotes, and that the edited lines had adapted to the knockout in different ways. To dissect the differences further, the CAGE-based transcriptional profiles of the *USP16* knockout homozygous, heterozygous and wild type clones were analyzed using BioLayout network analysis software. A gene-to-gene analysis was performed with a relatively stringent Pearson correlation coefficient threshold of 0.92 and MCL clustering with an inflation coefficient of 2.0 (Figure 7A). This included 5640 nodes (representing CTSS, equivalent to gene promoters) making 109,930 edges (correlation of at least 0.92 between nodes). The majority of the nodes (4474 nodes, 105,618 edges) were in two distinct regions, which were associated with high expression either in THP-1 cells prior to treatment with PMA (monocytic cells), or in THP-1 cells after 24 h treatment (macrophage-like cells) (Figure 7A). The remaining nodes were in smaller elements of 2 to 154 nodes. Cluster lists for clusters discussed below are available in Supplementary Figure 2 and show groups of genes whose expression patterns were more similar to each other than to others in the analysis, as identified by the MCL clustering method. The largest cluster was Cluster 1 with 850 nodes (CTSS), which included the main *CSF1R* promoter and contained genes which were upregulated after differentiation (Figure 7B). Genes in this and other smaller clusters showing high expression 24 h after induction of differentiation tended to be associated with immunity, cell-cell adhesion and migration, neutrophil degranulation and the lysosome (analyzed by PANTHER – see Supplementary Figure 2), although there was a wide variety of GO terms found for these genes. The second most abundant cluster was Cluster 2 with 286 nodes, which contained genes highly expressed in monocytic THP-1 cells prior to differentiation (Figure 7B). A number of smaller clusters also showed this pattern and contained genes associated with cell division, the cell cycle and DNA replication and repair (Giotti et al., 2019). Notably cluster 6 (80 nodes) showed this pattern and contained the majority of the detected histone genes. There was little indication of an effect of absence of functional USP16 on genes in these clusters, showing that homozygous and heterozygous *USP16* knockout cells maintain most of the gene expression patterns of monocyte and macrophage-like wild type cells, consistent with the ability to phagocytose and differentiate shown above.

To understand how the edited clones are able to maintain these functions, we looked for clusters where the expression pattern was different from wild type. As we have seen previously for cell lines (Gazova et al., 2020) and inbred animals (Summers et al., 2020), there was considerable variation among replicates, reflected in clusters with idiosyncratic profiles usually dependent on a single sample (shown in Supplementary Table 1). The wild type replicates were all generated from the parent THP-1 clone 5, so these differences may reflect phenotypic drift or phenotypic heterogeneity of the parent clone. The edited samples represent different clonal lines, each of which may have responded to the effect of the knockout differently. In particular, Hom1 and its parental heterozygote HetC showed a different phenotype from the other heterozygote and homozygotes (Figures 3, 5). However, there were several clusters where there was a clear difference between the wild type replicates and the edited clones, enabling some insight into the mechanisms used by the cells to escape the effects of the absence of USP16.

Genes in Cluster 13 (38 nodes), Cluster 31 (15 nodes), Cluster 37 (13 nodes) and Cluster 44 (12 nodes) were high in most wild type replicates prior to PMA stimulation. These genes were low in wild type after 24 h of PMA treatment and low in all heterozygous and homozygous clones at both time points. Because of the small number of genes in the clusters, GO enrichment analysis was performed on the combined list of genes to increase the power. With Bonferroni correction, the only enriched terms were GO Cellular Component (GO CC) terms relating to the mitochondrial matrix. For ontology terms, the Bonferroni adjustment is considered conservative since the terms are not independent (PANTHER documentation^15^). Therefore we used a reduced stringency (raw *P*-value < 0.001) to look for possible enrichment. GO Biological Process (GO BP) terms relating to metabolic processes and mitochondrial functions were enriched at this level (Supplementary Table 2). In contrast, genes in Cluster 33 (14 nodes) were low in wild type prior to PMA stimulation and increased in wild type after treatment and were high in all homozygotes and heterozygotes at both time points. This group was too small for meaningful GO enrichment analysis. These clusters indicate genes which may be affected by the absence of USP16, either in the monocyte state or after the cells are triggered to differentiate. Notably, the group which is low in the edited clones contains the *SRPRB* gene (Cluster 13), which encodes a protein thought to be an interaction partner of USP16 (BioGRID database of protein, genetic and chemical interactions^16^).

There were several other clusters which contained genes that were differently expressed in wild type and the edited clones. The average expression of genes in Cluster 4 (140 nodes) was up-regulated 8 to 30-fold during differentiation of wild type THP-1 cells (Figure 7C) but only increased up to two-fold in the heterozygous and homozygous knockout clones. These genes are likely dependent on USP16 for expression. This cluster contained known interferon inducible genes, and a promoter for the gene encoding the key transcription factor IRF9. GO annotations for genes within this clusters were enriched for terms involved with interferon signaling and response to virus.

Cluster 11 (44 nodes) contained genes that were increased 6 to 8 fold in wild type 24 h after PMA treatment. These genes were also increased at 24 h in heterozygotes and homozygotes, but only 4- to 5-fold (HetC and Hom1) or 2- to 3-fold (HetdE and Homs 2-4) (Figure 7C). These genes appear to be impacted by the loss of USP16, even in the heterozygotes which had wild type levels of expression of *USP16* mRNA (Figure 2). There was no significant GO term enrichment for these genes using the stringent Bonferroni correction. As above, to gain further insight into the impact of USP16 absence, we therefore looked at GO terms using a less stringent uncorrected significance threshold of *P* < 0.001. At this significance threshold, enriched GO Biological Process (GO BP) terms were related to regulation of immune system processes and membrane transporter activity (Supplementary Table 2).

Cluster 19 (23 nodes) contained genes that were high only in HetC and Hom1 prior to PMA treatment (Figure 7D). Although there were no significant GO terms for this small cluster using the Bonferroni correction, genes included several involved in the cell cycle (*BUB1, ESPL1B*, and *FANCA*) (Giotti et al., 2019). This was reflected in the GO BP terms enriched at lower stringency (uncorrected *P* < 0.001) which included terms relating to chromosome separation (Supplementary Table 2). In all cases of genes with multiple promoters, a minor promoter was found in this cluster, suggesting that one way Hom1 escapes the lack of USP16 and maintains the ability to proliferate (shown by the high level of *MYB* seen in Figure 5B) is to up-regulate minor promoters of other genes to compensate.

Cluster 10 (52 nodes) was the reciprocal of Cluster 19, where average expression increased around 5-fold in most samples at 24 h, but in HetC and Hom1 these genes were increased 27- and 53-fold respectively (Figure 7D). Most promoters in this cluster were expressed only in these two samples. As for Cluster 19, where genes had more than one promoter in the analysis, promoters in this cluster were almost all minor (usually low expression) promoters, including the third promoter for *CSF1R.* There was no GO terms enrichment at the stringent Bonferroni corrected significance level, but there was enrichment for genes associated with the PANTHER *integrin signaling* pathway. To examine the adaptations of HetC and Hom1 we therefore looked at GO terms using an uncorrected significance threshold of *P* < 0.001, which showed enrichment for GO BP terms related to tumor necrosis factor production, negative regulation of cell death, positive regulation of macrophage proliferation, cytokine production, regulation of histone phosphorylation and morphogenesis (Supplementary Table 2).

Several other clusters contained genes that were up-regulated in at least some wild type samples and in HetC and Hom1 at 24 h. Genes in Cluster 15 (29 nodes) were up-regulated after PMA stimulation in three wild type samples and in Hom1 and HetC (Figure 7E). The cluster included the gene for CSF1, the major effector of macrophage differentiation. Cluster 16 genes were also up-regulated at 24 h in wild type, HetC and Hom1 (Figure 7E). Genes in Cluster24 were increased in two wild type samples at 24 h and to a lesser extent in HetC and Hom1 but not in the other homozygous and heterozygous cells (Figure 7E). GO enrichment analysis of the combined gene list from these clusters included terms related to mesodermal cell differentiation, inflammation and cell motility.

Since their phenotype showed greater difference to wild type, and their differentiation appeared less complete than Hom1 (Figures 3, 5), we also looked for clusters of genes that distinguished Hom2, Hom3 and Hom4 and the double-edited HetdE (from the same parent heterozygote) from wild type and HetC/Hom1. One small cluster (Cluster 42, 12 nodes; Figure 7F) contained genes that were increased in these cells prior to differentiation and slightly increased after 24 h with PMA. At the reduced stringency, enriched GO terms were related to biosynthetic processes and activation of plasma proteins involved in acute inflammation. However the major effects on these cells appeared to be reduction in expression of genes whose levels were maintained in HetC and Hom1.

## Discussion

This paper describes the impact of generating a homozygous inactivating mutation of the *USP16* gene in a high differentiation clone of the THP-1 acute monocytic leukemia cell line, using the CRISPR-Cas9 genome editing technique. The fact that we generated independent subclonal lines that proliferated in culture and were able to differentiate in response to PMA might imply that the many functions attributed to USP16 described in the introduction are redundant. However, our detailed analysis rather points to the ability of the leukemic line to adapt to the extreme selection pressure applied by the loss of USP16 through different compensatory pathways.

The generation of homozygous *USP16* knockout cells required two rounds of CRISPR-Cas9 mutagenesis. There were no homozygotes after the first round. After a second round using heterozygotes from the first round, 4 out of 67 viable clones were homozygous mutants, whereas 13 reverted back to wild type, presumably by homology-directed repair from the wild-type allele. This pattern strongly suggests that the survival and growth of homozygous *USP16*^–/–^ cells was compromised, consistent with the observation that *Usp16* knockout is embryonic lethal in mice (Yang et al., 2014). The THP-1 *USP16*^–/–^ survivors had likely up-regulated compensatory mechanisms (genetic or epigenetic) to overcome the deficiency. Since the surviving homozygotes had different phenotypes, depending on the heterozygote from which they originated, we conclude that different mechanisms were responsible for the viability of these clones. Aside from the loss of USP16, additional selection pressures derived from the harsh conditions of electroporation, FACS sorting and single cell cloning. For this reason, it is important to use controls that have also been subjected to the same process. We present results from heterozygote cells that had been through the same two rounds of CRISPR-Cas9 treatment. We also assessed doubly targeted cells which had reverted to wild type, for cell cycle characteristics; they showed no difference from the parental wild type cells.

Arguably, selection pressures similar to those of CRISPR-Cas9 treated cultures occur during the evolution of a cancer *in vivo* (Fortunato et al., 2017). Although our starting population was a clonal line selected for high differentiation potential (Suzuki et al., 2009), THP-1 cells have deficient mutation repair (Bauer et al., 2011) and the original clonal population is likely by now to contain divergent lines characterized by different mutations. The heterozygous mutant lines from the first round may have been derived from cells that had accumulated other mutations or epigenetic modifications that compensated at least in part for the loss of *USP16* and allowed the survival of the homozygotes after the second round of selection.

The simple MTT viable cell assay suggested that three of the homozygous mutant clones (Hom2, Hom3, and Hom4) grew marginally more slowly than the parent (Figure 3B) but this was not supported by cell cycle analysis (Figure 3C). There were some differences in down-regulation of *MYB* and up-regulation of *CSF1R* between wild type and homozygous clones (Figure 5). However, in overview the loss of USP16 did not prevent THP-1 cells from proliferating or differentiating to become adherent phagocytic macrophages in response to PMA (Figure 3A).

To dissect the transcriptome of THP-1 wild type cells with the *USP16* knockout clones we used CAGE, a promoter based approach that provides expression levels by capturing mRNA with the 5′ modified guanine cap (Balwierz et al., 2009; Forrest et al., 2014). The CAGE data (Figure 2B) confirmed reduction of *USP16* mRNA in the homozygous knockout clones both before and after PMA treatment, presumably due to nonsense-mediated decay. Combined with the lack of effect of heterozygous mutation on USP16 protein (Figure 2C) the data suggest there is dosage compensation.

The H2AK119 deubiquitinases are collectively involved in cell cycle progression and DNA repair (Atanassov et al., 2011; Chen et al., 2015; Aquila and Atanassov, 2020), and the most obvious potential mechanism to escape the impacts of loss of USP16 would be to up-regulate related deubiquitinases. Other studies have observed interdependence of deubiquitinase mRNA levels. For example, *USP12* down-regulation resulted in *USP46* up-regulation (Joo et al., 2011). Indeed, mRNA encoding USP12, BAP1 and MYSM1 was increased in all USP16-deficient lines and Hom1 also had increased *USP21* mRNA, encoding another H2AK119 deubiquitinase. *USP3* and *USP22* mRNA showed distinct patterns of down-regulation by PMA in the different homozygous lines. Both over- and under-expression of *USP3* have been shown to have effects consistent with tumorigenesis. For example, USP3 promoted proliferation in a number of cancers (Fang et al., 2018; Wu et al., 2019; Das et al., 2020; Li et al., 2020; Liao et al., 2020) while other reports show that depletion of USP3 can increase the incidence of spontaneous tumors (Lancini et al., 2016), promote metastasis (Wang et al., 2017), inhibit leukemia cell differentiation (Chae et al., 2019), and accelerate degradation of TP53 leading to enhanced proliferation and transformation (Fu et al., 2017).

Our transcriptional network analysis of individual clones in the presence and absence of PMA complements our recent high-density time course analysis of the differentiation response in the parent line (Gazova et al., 2020). In effect it is a perturbation analysis in which intrinsic plasticity and genetic-epigenetic instability of THP-1 and clonal heterogeneity, as well as the specific loss of USP16, all contribute to the phenotype. Genes in Clusters 4 and 11 were reduced in all edited cells, both homozygotes and heterozygotes, and were associated with interferon signaling and immune responses. Transfection with plasmid DNA to generate mutations activates the AIM2 inflammasome and cGAS pathways in THP-1 cells (Burckstummer et al., 2009; Paijo et al., 2016) leading to interferon induction and inhibition of proliferation as well as cell death. It is very likely that the generation of mutants selects against interferon responsiveness. Conversely, other clusters of genes were greatly increased in the edited cells, albeit not tightly correlated with *USP16* genotype. HetC and Hom1, where the phenotype was closer to wild type, up-regulated the key regulator, *MYB* (Suzuki et al., 2009) which may overcome a partial block on proliferation caused by the loss of USP16.

Despite the subtle differences in gene expression amongst the clones, irrespective of the loss of USP16 and the apparent loss of interferon signaling, all of the lines were able to undergo macrophage-specific cellular differentiation and some degree of growth inhibition in response to PMA, indicated by the coordinated regulation of transcripts within Clusters 1 and 2 respectively. They each expressed the macrophage-specific transcription factor *SPI1* (encoding PU.1) constitutively with a similar signal from the upstream enhancer identified previously (Suzuki et al., 2009; Gazova et al., 2020). The 24 h time point occurs before induction of surface markers such as ITGAM (CD11B) and APOE, but the inducible genes in Cluster 1 include *SPP1* (encoding osteopontin) and *CSF1R*, both of which were highlighted in earlier analyses (Suzuki et al., 2009) as well as genes encoding macrophage surface receptors and lysosomal enzymes. Each clone showed similar massive induction of the key cell cycle inhibitor, *CDKN1A* (p21WAF) highlighted previously (Gazova et al., 2020) and Cluster 2, down-regulated in all lines, contains numerous S-phase cell cycle-related transcripts including *CDK2, E2F1*, and *PCNA.*

A limitation of this study is that it used an immortalized cell line derived from an acute myeloid leukemia 40 years ago. The compensation phenomenon we have described is specific to cells under the intense selection of USP16 deletion. Generation of homozygous knockout clones was a rare event in our study, with strong selection in the second targeting for cells that had reverted to wild type. To better understand the role of USP16 and possible compensatory mechanisms, cells from newly diagnosed tumors could be examined. In particular, changes in expression of the histone deubiquitinases over the evolution of the tumor and during relapses would reveal whether the mechanism proposed here is also found in primary tumors. There are some examples of apparent down-regulation of USP16 in cancer (Fernandez et al., 2004; Gelsi-Boyer et al., 2008; Qian et al., 2016) but *USP16* mutation is rare in primary cancers (data from TCGA Project^17^) which may be consistent with its important role in the cell cycle and DNA repair, and a reason why it has been considered as a cancer drug target. The gene is actually expressed ubiquitously in every tumor cell line (leukemias, sarcomas, adenocarcinomas) and proliferating primary cell population that was analyzed in the large FANTOM5 project (Forrest et al., 2014). A study of those rare cancers which have inactivating *USP16* mutations would reveal whether the compensatory mechanisms proposed here are also operating *in vivo*.

In conclusion, this communication is a case study in leukemic cell adaptation. The original functional analysis of USP16 (Joo et al., 2007) reported a 3-fold slowing of proliferation, M phase arrest and increased basal H2A ubiquitination in HeLa cells with a stable 90% knock down of the USP16 protein. On that basis one might consider USP16 as a target for cancer chemotherapy and indeed that has been proposed (Harrigan et al., 2018). Our study shows clearly that USP16 is potentially redundant and with sufficient selection pressure, leukemic cells would give rise to escape mutants that are resistant to USP16 inhibitors.

## ^‡^Orcid:

Iveta Gažová

orcid.org/0000-0002-0787-9463

Lucas Lefevre

orcid.org/0000-0003-0925-7411

Stephen J. Bush

orcid.org/0000-0001-9341-2562

Rocio Rojo

orcid.org/0000-0001-9686-3377

David A. Hume

orcid.org/0000-0002-2615-1478

Andreas Lengeling

orcid.org/0000-0002-7992-2563

Kim M. Summers

orcid.org/0000-0002-7084-4386

## Data Availability Statement

The datasets presented in this study can be found in an online repository at https://www.ebi.ac.uk/ena, PRJEB43087. The results can be visualised using the ZENBU browser at http://fantom.gsc.riken.jp/zenbu/gLyphs/#config=Gazova_USP16KO.

## Author Contributions

KS, AL, and DH designed and supervised the project. IG, LL, and RR performed the laboratory work. IG, SB, and KS performed the bioinformatic analysis. IG and KS wrote the manuscript. All authors read and approved the manuscript.

## Conflict of Interest

The authors declare that the research was conducted in the absence of any commercial or financial relationships that could be construed as a potential conflict of interest.

## Footnotes

1 https://fantom.gsc.riken.jp/zenbu/, accessed April 2021

2 https://www.cancer.gov/tcga, accessed April 2021

3 http://crispr.mit.edu

4 https://www.ensembl.org

5 http://web.expasy.org/translate/

6 http://fantom.gsc.riken.jp/zenbu/

7 http://tmcalculator.neb.com

8 https://technelysium.com.au/wp/chromas/

9 http://primer3.ut.ee/

10 https://zenbu-wiki.gsc.riken.jp/zenbu/wiki/index.php/OSCtable

11 http://fantom.gsc.riken.jp/zenbu/gLyphs/#config=Gazova_USP16KO

12 http://biolayout.org

13 https://www.rdocumentation.org/packages/edge/versions/2.4.2

14 http://geneontology.org/, Go Ontology database DOI: 10.5281/zenodo.4081749 Released 9 October 2020

15 pantherdb.org/tips/tips_bonferroni.jsp, accessed February 2021

16 https://thebiogrid.org, accessed February 2021

17 https://www.cancer.gov/about-nci/organization/ccg/research/structural-genomics/tcga, accessed April 2021

## Supplementary Material

Supplementary Figure 1 Graph size vs correlation threshold for BioLayout analysis.

Supplementary Figure 2 Image of CAGE tags mapping to *USP16* taken from the ZENBU viewer.

Supplementary Table 1 Clusters of coexpressed genes.

Supplementary Table 2 GO term enrichment analysis for selected clusters.
