# Supplementary material for: CRISPR-Cas9 Editing of Human Histone Deubiquitinase Gene USP16 in Human Monocytic Leukemia Cell Line THP-1
Source: Front Cell Dev Biol. 2021 May 31;9:679544. doi: 10.3389/fcell.2021.679544 (PMC8203323; doi:10.3389/fcell.2021.679544)
Supplement: Supplementary Figure 1 — Graph size vs correlation threshold for BioLayout analysis. [file Data_Sheet_1.pdf]

Supplementary Figures

CRISPR-Cas9 editing of human histone deubiquitinase gene *USP16* in human monocytic leukemia cell line THP-1

Iveta Gažová, Lucas Lefevre, Stephen J Bush, Rocio Rojo, David A Hume, Andreas Lengeling, Kim M Summers

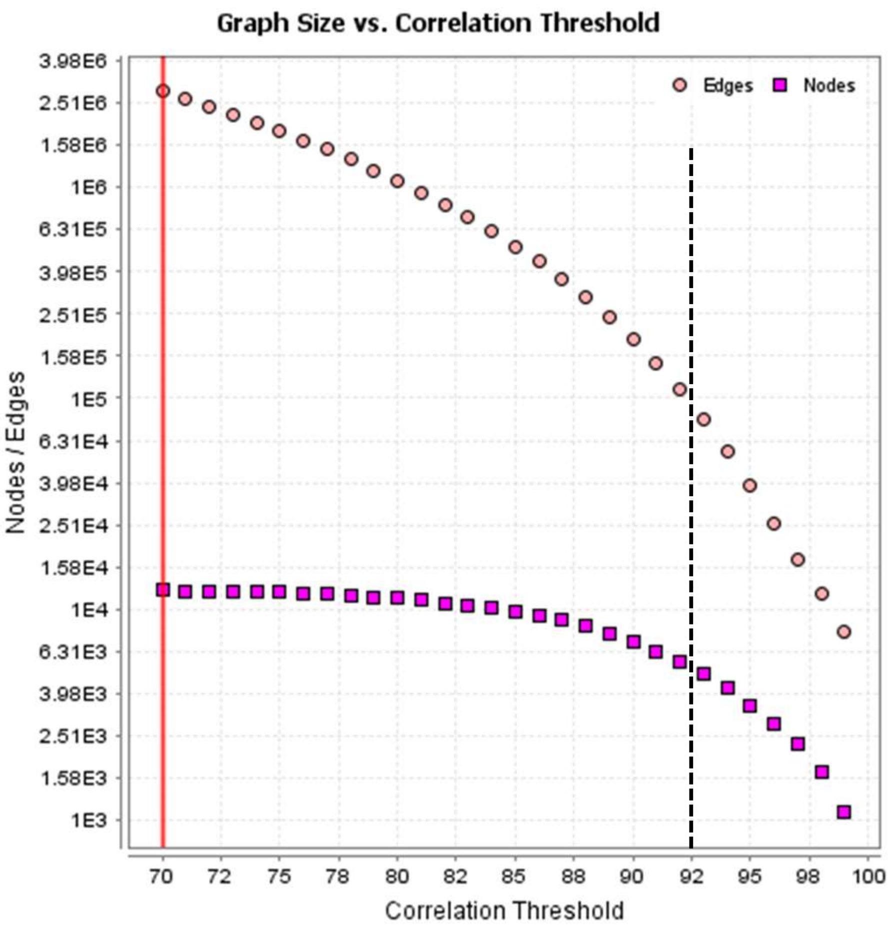

Figure S1. Graph size vs correlation threshold for BioLayout analysis
The graph illustrates the relationship between the number of nodes and the number of edges (Freeman et al., 2007; Theocharidis et al., 2009). The correlation threshold chosen for the analysis was 0.92, indicated by the dotted black line. This included 5,640 nodes making 109,930 edges.

A

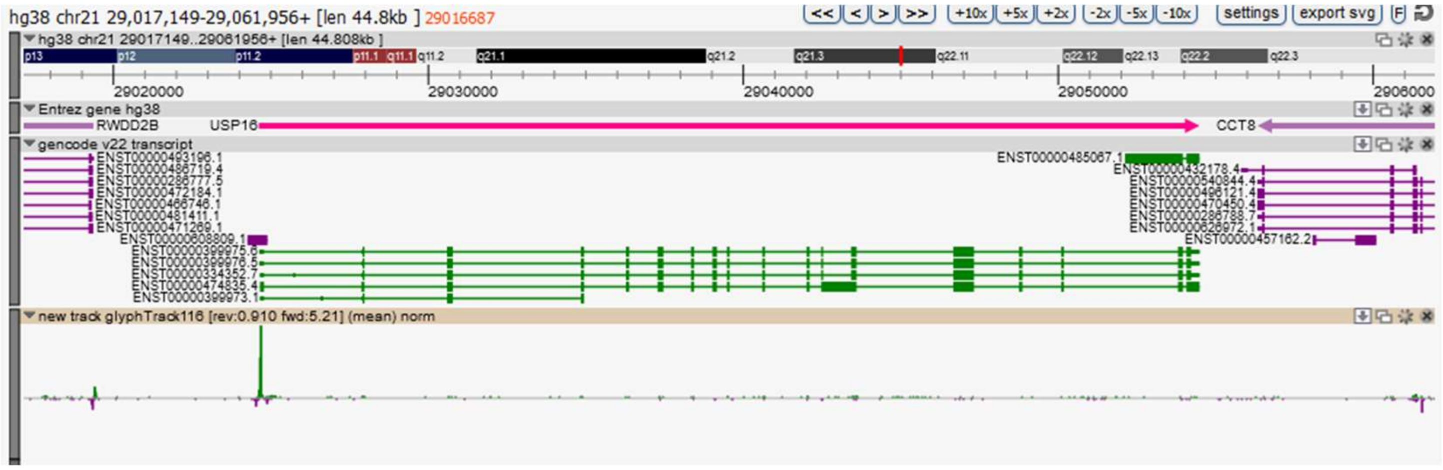

B

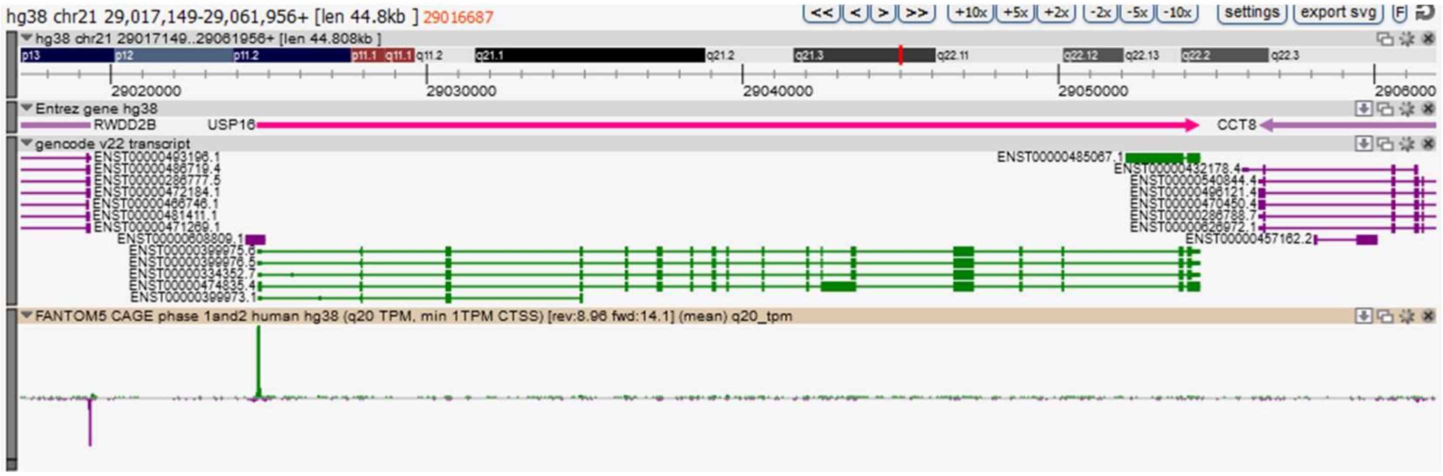

Figure S2. Image of CAGE tags mapping to *USP16* taken from the ZENBU viewer.

In **A** and **B**, the upper tracks show the Entrez gene and Gencode v22 transcripts for *USP16.* The lower track shows the CAGE tags mapped to the region. Green indicates tags mapped the forward direction; purple indicates tags mapped to the reverse direction. *USP16* reads in the forward direction and the promoter is indicated by the peak of green tags on the left of the images. There is no indication of a second promoter for this gene.

A. CAGE tags for the *USP16* edited cells and wild type THP-1 cells.

B. CAGE tags for the whole FANTOM5 dataset for human. This set consists of approximately 1,000 tissues, primary cells, cancers and cell lines in varying stages of differentiation and activation (Forrest et al., 2014; Arner et al., 2015).

References for Supplementary Figures

Arner, E., et al. (2015). Transcribed enhancers lead waves of coordinated transcription in transitioning mammalian cells. Science. 347, 1010-1014. 10.1126/science.1259418

Forrest, A.R., et al. (2014). A promoter-level mammalian expression atlas. Nature 507, 462-470. 10.1038/nature13182

Freeman, T.C., Goldovsky, L., Brosch, M., Van Dongen, S., Maziere, P., Grocock, R.J., Freilich, S., Thornton, J., and Enright, A.J. (2007). Construction, visualisation, and clustering of transcription networks from microarray expression data. PLoS Comput Biol 3, 2032-2042.10.1371/journal.pcbi.0030206

Theocharidis, A., Van Dongen, S., Enright, A.J., and Freeman, T.C. (2009). Network visualization and analysis of gene expression data using BioLayout Express3D. Nat Protoc 4, 1535-1550. 10.1038/nprot.2009.177
